# A human model of bilateral pulmonary vein sampling to assess the effects of one-lung ventilation on neutrophil function

**DOI:** 10.1371/journal.pone.0271958

**Published:** 2022-07-26

**Authors:** Wendy Funston, Marie-Hélène Ruchaud-Sparagano, Jonathan Scott, Jason Powell, Faye A. H. Cooles, Lauren Shelmerdine, Cliona McDowell, Denis O’Leary, Karen L. Booth, Stephen C. Clark, Simon J. Ledingham, Anthony J. Rostron, John H. Dark, A. John Simpson

**Affiliations:** 1 Faculty of Medical Sciences, Newcastle University, Newcastle upon Tyne, United Kingdom; 2 Health Education England North East, Newcastle upon Tyne, United Kingdom; 3 Northern Ireland Clinical Trials Unit, The Royal Hospitals, Belfast, Northern Ireland, United Kingdom; 4 Department of Cardiothoracic Anaesthesia, Newcastle upon Tyne Hospitals NHS Foundation Trust, Newcastle upon Tyne, United Kingdom; 5 Department of Cardiothoracic Surgery, Newcastle upon Tyne Hospitals NHS Foundation Trust, Newcastle upon Tyne, United Kingdom; 6 Faculty of Health and Life Sciences, University of Northumbria, Newcastle upon Tyne, United Kingdom; Stanford University School of Medicine, UNITED STATES

## Abstract

**Background:**

Neutrophil activation drives lung complications after cardiopulmonary bypass (CPB). Evidence suggests the healthy, ventilated lung may beneficially re-condition pro-inflammatory neutrophils. However, evidence in humans is lacking, due to a paucity of good models. CPB with simultaneous central venous and bilateral pulmonary vein sampling provides an opportunity to model effects of one-lung ventilation. The study’s primary objectives were to establish a model of intra-operative, bilateral pulmonary vein sampling and to determine whether neutrophil function differed after passing through inflated or deflated lungs.

**Methods:**

Seventeen patients having “on pump” coronary artery bypass grafting (CABG) with one-lung ventilation (in two cohorts with tidal volume 2ml kg^-1^ and FiO_2_ 0.21, or tidal volume 4 ml kg^-1^ and FiO_2_ 0.5 respectively) were recruited. Cohort 1 consisted of 9 patients (7 male, median age 62.0 years) and Cohort 2 consisted of 8 male patients (median age 65.5 years). Recruitment was via prospective screening of scheduled elective and non-elective CABG procedures with cardiopulmonary bypass. Each patient had five blood samples taken—central venous blood pre-operatively; central venous blood pre-CPB; central venous blood post-CPB; pulmonary venous blood draining the ventilated lung post-CPB; and pulmonary venous blood draining the deflated lung post-CPB. Neutrophil phagocytosis and priming status were quantified. Plasma cytokines were measured.

**Results:**

Phagocytosis and priming were not significantly different in neutrophils returning from the ventilated lung as compared to the non-ventilated lung. Plasma IL-6, IL-8 and IL-10 were significantly elevated by CPB.

**Conclusions:**

The intra-operative, bilateral pulmonary vein sampling model provides unique opportunities to assess biological effects of interventions to one lung, with the other lung acting as an internal control. Single-lung ventilation during CPB had no significant effects on neutrophil function.

## Introduction

Prior exposure to inflammatory mediators “primes” neutrophils, significantly lowering the threshold for activation by subsequent stimuli [1]. Priming is a key step in achieving maximal neutrophil activation and a prerequisite for neutrophil-mediated tissue injury [2]. Primed neutrophils are retained in the human lung [3]. Emerging evidence suggests the healthy human lung may modulate circulating neutrophil function, for example by de-priming neutrophils [4]. Study in this area has been hampered by the inability to compare neutrophil function immediately after passage through the human lung. Cardiopulmonary bypass (CPB), routinely used in cardiac surgery, provides an opportunity to study neutrophils in this way, as an intervention can be selectively applied to one lung, while the other serves as a control, and blood from the pulmonary veins draining each can be sampled separately.

It is not known whether adequate ventilation of the lung is required to restore normal function to neutrophils, or whether collapse or impaired ventilation of the lung may impair restoration of neutrophil function. The primary objectives of this preliminary study were therefore to establish and describe a model of intra-operative bilateral pulmonary vein sampling during one-lung ventilation (OLV), and to assess whether neutrophil function was impaired by passage through a deflated lung. Secondary objectives were to determine whether any clear adverse clinical outcomes were associated with the model.

Pulmonary complications occur frequently following cardiac surgery with CPB, with neutrophil dysfunction implicated as a cause of the associated morbidity and mortality [5–8]. In describing the intra-operative bilateral pulmonary vein sampling model, an additional aim was to promote further research into mechanisms underlying post-operative complications.

## Materials and methods

### Patients and study design

This was an exploratory, observational preliminary cohort study carried out in a UK teaching hospital (Freeman Hospital, Newcastle upon Tyne Hospitals NHS Foundation Trust) between May 2016 and May 2017. Electronic surgical lists of patients scheduled for elective or non-elective CABG with CPB were screened prospectively. Eligible patients were offered verbal and written information on the study.

Patients were eligible for inclusion if they were >18 years of age and were awaiting either elective or non-elective on-pump coronary artery bypass graft procedures at our institution during the study period. Exclusion criteria comprised: age <18 years; regular immunosuppressant medications (prednisolone in a maintenance dose up to 7.5 mg per day was permitted); repeat or re-do CABG; CABG procedures requiring additional surgical interventions (e.g., valve replacements); absence of informed written consent; and participation in a clinical trial that may affect leukocyte function. The specified exclusion criteria ensured that the study population was as representative as possible of the population of patients undergoing this procedure. Patients’ medical notes were reviewed, and clinical data recorded. The Study was approved by the North East-York Research Ethics Committee (15/NE/0319) and was registered with ISRCTN (ISRCTN70523327). Informed written consent was obtained from all participants. The Newcastle upon Tyne Hospitals NHS Foundation Trust acted as sponsor.

### Anesthetic and ventilation procedures

Routine anesthetic induction procedures were followed for each patient. Following insertion of peripheral venous and radial artery cannulae patients were pre-oxygenated with FiO_2_ 1.0. Induction and maintenance of anesthesia was at the discretion of the anesthetist. Participants were intubated and mechanically ventilated with a single lumen endo-tracheal tube according to a standardized lung protective ventilation protocol (see supporting information section: S1 Protocol). When CPB was commenced, a Cohen endobronchial blocking balloon (Cook Medical, Indiana, USA), which had been placed in the left main bronchus following intubation, was inflated, ensuring ventilation of the right lung, but not the left, throughout CPB.

Two cohorts were assessed, to determine effects of different tidal volumes in the inflated right lung—cohort 1: volume-controlled ventilation, tidal volume 2 mL kg^-1^ of ideal body weight (IBW), FiO_2_ 0.21, positive end-expiratory pressure (PEEP) 5 cmH_2_O, 7 ventilations per minute, and cohort 2: volume-controlled ventilation, tidal volume 4 mL kg^-1^ IBW, FiO_2_ 0.5, PEEP 5 cmH_2_O, 7 ventilations per minute (see supporting information section: S1 Protocol).

Following completion of CABG, the left bronchus-blocking balloon was deflated and both lungs re-ventilated according to a standardized lung protective ventilation protocol (see supporting information section: S1 Protocol), prior to the patient being weaned off CPB.

### Surgical procedures

Intravenous tranexamic acid (1 gram) was administered prior to skin incision and median sternotomy (as per standard protocol for major cardiac operations in our institution to reduce post operative bleeding). The left internal mammary artery was dissected down with simultaneous saphenous vein harvesting from the leg. Intravenous unfractionated heparin was administered and the aorta cannulated with an armored, angled cannula. An armored two-stage venous cannula was placed in the right atrium through the appendage. CPB was commenced with systemic cooling to 34°C. The extracorporeal circuit consisted of a roller pump (Stockert S5 model, LivaNova, London, UK) and membrane oxygenator (CAPIOX^®^RX, Terumo Cardiovascular Group, Ann Arbor, Michigan, USA) for all patients.

A mean arterial pressure target of 50–80 mmHg was set for each patient. Pump-flow was adjusted on an individual basis, guided by regular measurements of mixed venous oxygen saturations. After aortic cross-clamping, cold blood cardioplegia was administered to the aortic root via a separate cannula and myocardial protection augmented with topical cold saline and ice slush. Following completion of all vascular anastomoses the aortic cross-clamp was removed and, with normothermia achieved, CPB was discontinued over approximately 5 minutes. Protamine sulphate was administered to reverse the effects of heparin followed by decannulation of the heart. Atrial and right ventricular pacing wires were placed prophylactically prior to mediastinal and left pleural drain placement and chest closure.

The surgical and anesthetic approaches for CABG described above are widely practiced in cardiothoracic centers throughout the world, and so the study population is considered a representative cohort for a larger population.

### Blood sampling protocol

Five venous blood samples (each 25 mL) were obtained as described in Table 1.

**Table 1:** Blood sampling protocol.

Sample	Description	Volume
**1**	**Venous blood**:	25 mL
	Taken from a central line immediately following insertion (i.e. after induction of anesthesia)	
**2**	**Venous blood**:	25 mL
	Taken from a central line just before unfractionated intravenous heparin administered (i.e. just prior to the initiation of CPB)	
**3**	**Venous blood**:	25 mL
	Taken from a central line:	
	after grafts have been completed, normal ventilation resumed, taken off CPB and protamine given, but before chest closure	
**4**	**Venous blood**:	25 mL
	Taken from a right pulmonary vein (i.e. sampling blood that has been through the ventilated lung) at same time as sample 3	
**5**	**Venous blood**:	25 mL
	Taken from a left pulmonary vein (i.e. sampling blood that has been through the deflated lung) at same time as sample 3	

### Neutrophil Isolation

Neutrophils were isolated simultaneously from each blood sample using dextran sedimentation and fractionation over discontinuous Percoll density gradients [9]. Isolated cells were counted using a hemacytometer. Cytospin slides were prepared and stained with Giemsa to determine neutrophil purity. Only samples with ≥90% neutrophil purity (as assessed by morphological analysis) were included.

### Neutrophil function

Neutrophil phagocytic capacity was assessed using two independent methods: a light microscopic analysis of isolated neutrophils using zymosan particles pre-opsonized using autologous serum [10, 11], and a flow cytometric analysis of whole blood using pHrodo^™^ Green *Staphylococcus aureus* Bioparticles^®^ (Thermofisher, MA, USA) [12]. Neutrophil priming was assessed by quantifying expression of the cell surface markers CD11b and CD62L using a whole blood flow cytometric method as previously described [4, 13].

### Neutrophil viability

The percentage of live, early apoptotic, late apoptotic and necrotic neutrophils was assessed by flow cytometry using allophycocyanin (APC)-conjugated annexin V (AnV) and propidium iodide (PI) [13].

### Quantification of inflammatory mediators

Cytokine quantification was performed on platelet-free plasma using a human V-PLEX pro-inflammatory kit (Meso Scale Diagnostics, MD USA) according to the manufacturer’s instructions.

### Clinical data

Clinical data were collected from all study participants’ medical records. Clinical outcome data for study participants from cohort 1 and cohort 2 were compared to age- and sex-matched controls who had undergone CABG procedures during the study period but who had not been recruited to our study. Control data were sourced from the hospital’s cardiothoracic registry by a member of staff independent of the study and included the following: total CPB time, time to extubation, duration of Intensive Care Unit (ICU) stay, total blood loss from mediastinal and pleural drains and post-operative chest radiograph reports. Control data was collected to compare clinical outcome data of patients in our study with patients who were not recruited to our study, primarily to monitor for any adverse safety signals given the novelty of our model.

### Sample size and statistical analysis

The primary outcome measure was neutrophil phagocytosis, assessed by the zymosan ingestion assay. Previous data indicated a normal distribution, mean 80.5%, standard deviation 7.7% [14]. Severe neutrophil dysfunction equates to <50% phagocytosis [15]. Considering a fall in phagocytosis of >2 standard deviations (SD) to be significant, a paired model (samples 4 versus 5) with alpha 0.05 and power 0.9 required 6 patients per cohort. To account for drop out or technical laboratory issues, sample sizes of 8–9 patients were proposed.

In the study, data proved not to be normally distributed and non-parametric statistical tests were applied. The Friedman test with Dunn’s post-hoc multiple comparisons was used for comparing values between the baseline blood sample (sample 1) and samples 2–5. Wilcoxon’s matched pairs signed-rank test evaluated differences between samples 4 and 5. The Mann-Whitney U test was used to compare clinical and perioperative parameters between patient cohorts and the control group. Patients with incomplete datasets were excluded from statistical analysis (i.e. when at least one of the five blood samples were not obtained or where laboratory data for one or more samples were not available). Data are represented as median values ± interquartile range (IQR) and were analyzed using GraphPad Prism≅ version 7.01 (GraphPad software, La Jolla, California, USA).

## Results

Consort diagrams for cohorts 1 and 2 are in the supporting information section (S1 and S2 Figs). Nine patients were recruited to cohort 1 and 8 to cohort 2. Clinical characteristics are shown in Table 2.

**Table 2:** Demographics and baseline clinical characteristics for the first and second cohorts.

	First cohort	Second cohort
**N**	9	8
**Male**	7	8
**Age (years)**	62.0 (57.5–78.0)	65.5 (60.3–74.5)
**Height (cm)**	171.4 (162.0–178.0)	173.0 (166.3–175.8)
**Weight (kg)**	77.0 (70.5–89.8)	84.2 (76.5–101.4)
**Ideal body weight (kg)**	67.2 (56.0–73.2)	68.7 (63.7–71.2)
**Smoking status (number of patients)**		
**Current smoker**	0	0
**Ex-smoker**	4	5
**Never smoker**	5	3
**Lung function**		
**FEV_1_ (liters)**	2.8 (2.3–3.9)	2.9 (2.5–3.0)
**FVC (liters)**	4.2 (3.2–5.0)	4.1 (3.1–4.3)
**FEV_1_ predicted (%)**	97.0 (93.0–119.5)	93.5 (80.5–101.5)
**FEV_1_/FVC (%)**	79.0 (68.0–80.5)	73.5 (69.0–82.5)
**Logistic EUROscore** ^§^	1.2 (1.0–4.3)	2.4 (1.6–4.5)
**Urgency of procedure (number of patients)**		
**Elective**	8	7
**Urgent**	1	1
**Co-morbidities (number of patients)**		
**Cardiovascular**		
**Previous MI**	6	5
**Previous PCI**	3	3
**Hypertension**	7	6
**Reduced EF (≤40%)**	1	2
**Permanent Atrial Fibrillation**	0	1
**Respiratory**		
**Asthma**	3	1
**COPD**	0	1
**Pleural plaques**	0	1
**Other**		
**Type 2 diabetes mellitus**	3	3
**GERD**	3	1
**Osteoarthritis**	3	2
**Liver cirrhosis**	1	0
**PMR**	1	0
**Chronic kidney disease (eGFR < 60)**	0	1
**Transient ischemic attack**	0	1

Data are presented as median (interquartile range).

^§^ Logistic Euroscore: a formula used to predict the risk of death after a cardiac operation [16].

Abbreviations: COPD, chronic obstructive pulmonary disease, EF: ejection fraction, FEV_1_: forced expiratory volume in 1 second, FVC: forced vital capacity, GERD: gastro-esophageal reflux disease, MI: myocardial infarction, PCI: percutaneous coronary intervention, PMR: polymyalgia rheumatica, eGFR: estimated glomerular filtration rate.

All patients underwent on-pump CABG procedures with saphenous vein harvesting. All five blood samples were obtained in eight patients in cohort 1 and six patients in cohort 2. For three patients (one in cohort 1 and two in cohort 2) the left pulmonary vein sample (deflated lung) was not obtained. Operative data are shown in supporting info section (S1 Table).

### Neutrophil function

**Fig 1:**
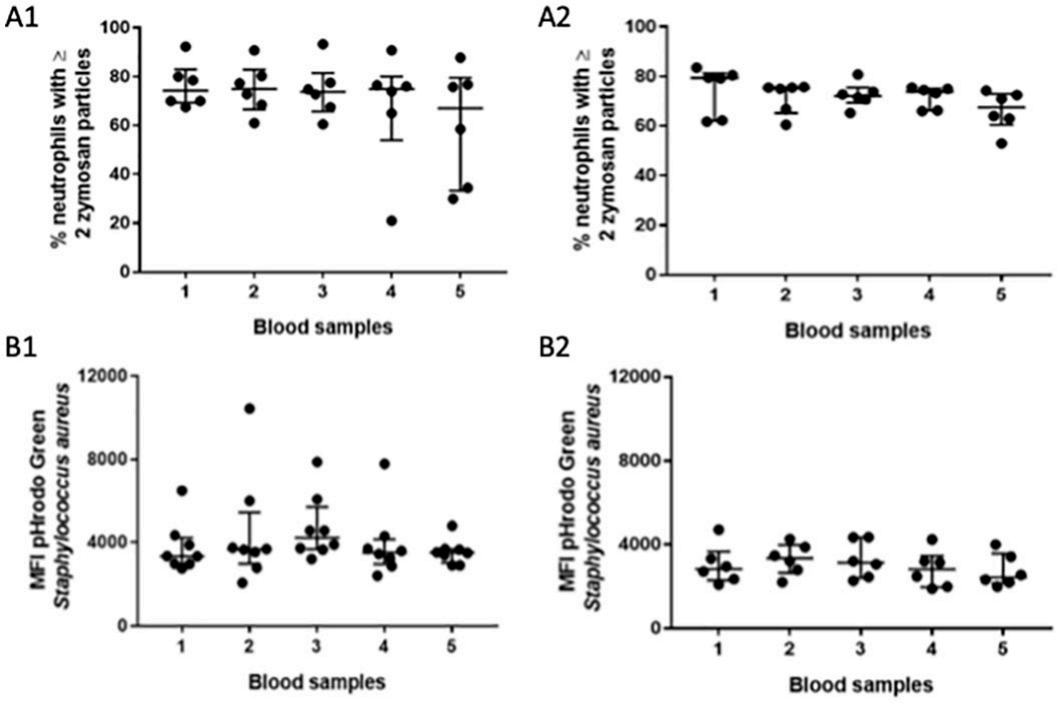
Blood neutrophil phagocytosis. Phagocytosis was quantified on neutrophils isolated from whole blood using a light microscopic assay of opsonized zymosan uptake: [A1] n = 6 (cohort 1) and [A2] n = 6 (cohort 2). Separately, phagocytosis was quantified whole blood using flow cytometric evaluation of internalized of pHrodo green *Staphylococcus aureus* bioparticles: [B1] n = 8 (cohort 1) and [B2] n = 6 (cohort 2). Data are presented on scatter plots with median (middle bar) and interquartile range (whiskers). The Friedman test with Dunn’s post-hoc multiple comparisons was used to compare values between sample 1 and samples 2–5. Wilcoxon’s matched pairs signed rank test was used to compare the right, ventilated lung (sample 4) and the left, deflated lung (sample 5). Abbreviations: MFI: median fluorescence intensity.

**Fig 2:**
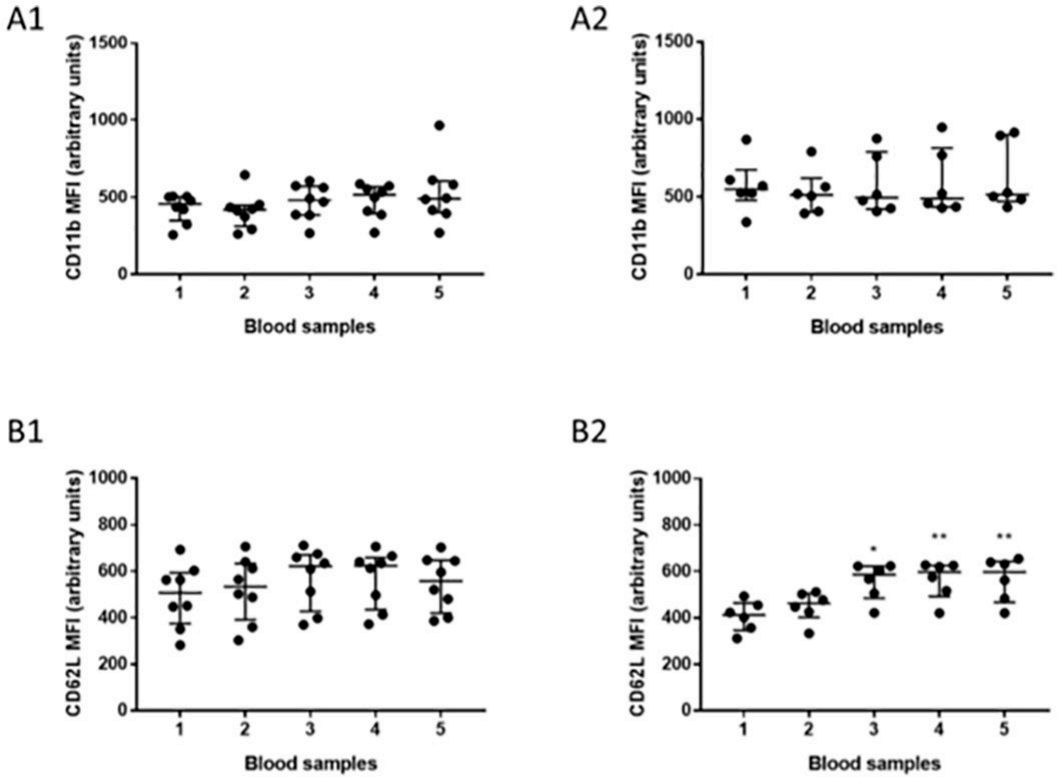
Neutrophil priming status. Data are presented as scatter plots with median (middle bar) and interquartile range (whiskers). Left-sided graphs represent cohort 1 and right-sided graphs cohort 2. [A1] and [A2] MFI of CD11b. [B1] and [B2] MFI for CD62L. The Friedman test with Dunn’s post-hoc multiple comparisons was used to compare values between sample 1 and samples 2–5. Wilcoxon’s matched pairs signed rank test was used to compare samples 4 and 5. *p<0.05, **p<0.005. (Cohort 1: n = 8. Cohort 2: n = 6). Abbreviations: MFI: median fluorescence intensity.

There was no significant change in neutrophil phagocytosis in either cohort (Fig 1). Primed neutrophils were identified by up-regulation of CD11b expression and down-regulation of CD62L expression. No differences in priming were observed with the exception that, in cohort 2, CD62L expression was significantly higher in the post-CPB samples compared to the pre-CPB samples (Fig 2).

### Neutrophil viability

**Fig 3:**
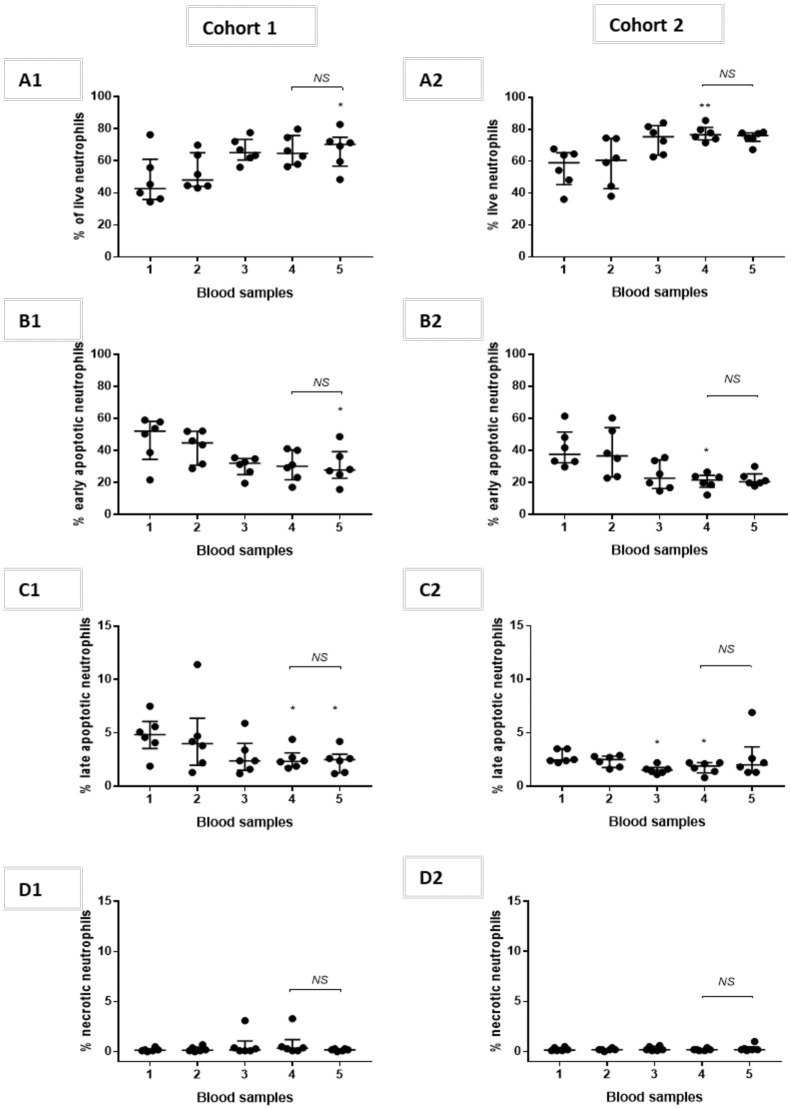
Neutrophil apoptosis. Data are presented as scatter plots with median (middle bar) and interquartile range (whiskers). Left-sided graphs represent cohort 1 (n = 6) and right-sided graphs cohort 2 (n = 6). [A1] and [A2] percentage of live neutrophils, [B1] and [B2] percentage of early apoptotic neutrophils, [C1] and [C2] percentage of late apoptotic neutrophils and [D1] and [D2] percentage of necrotic neutrophils. The Friedman test with Dunn’s post-hoc multiple comparisons was used to compare values between sample 1 and samples 2–5. Wilcoxon’s matched pairs signed rank test compared samples 4 and 5. Abbreviations NS: non-significant.

A trend towards more live neutrophils was observed in post-CPB samples compared to pre-CPB samples in both cohorts (Fig 3). The proportion of apoptotic neutrophils leaving the inflated and deflated lung appeared similar.

### Plasma cytokines

**Fig 4:**
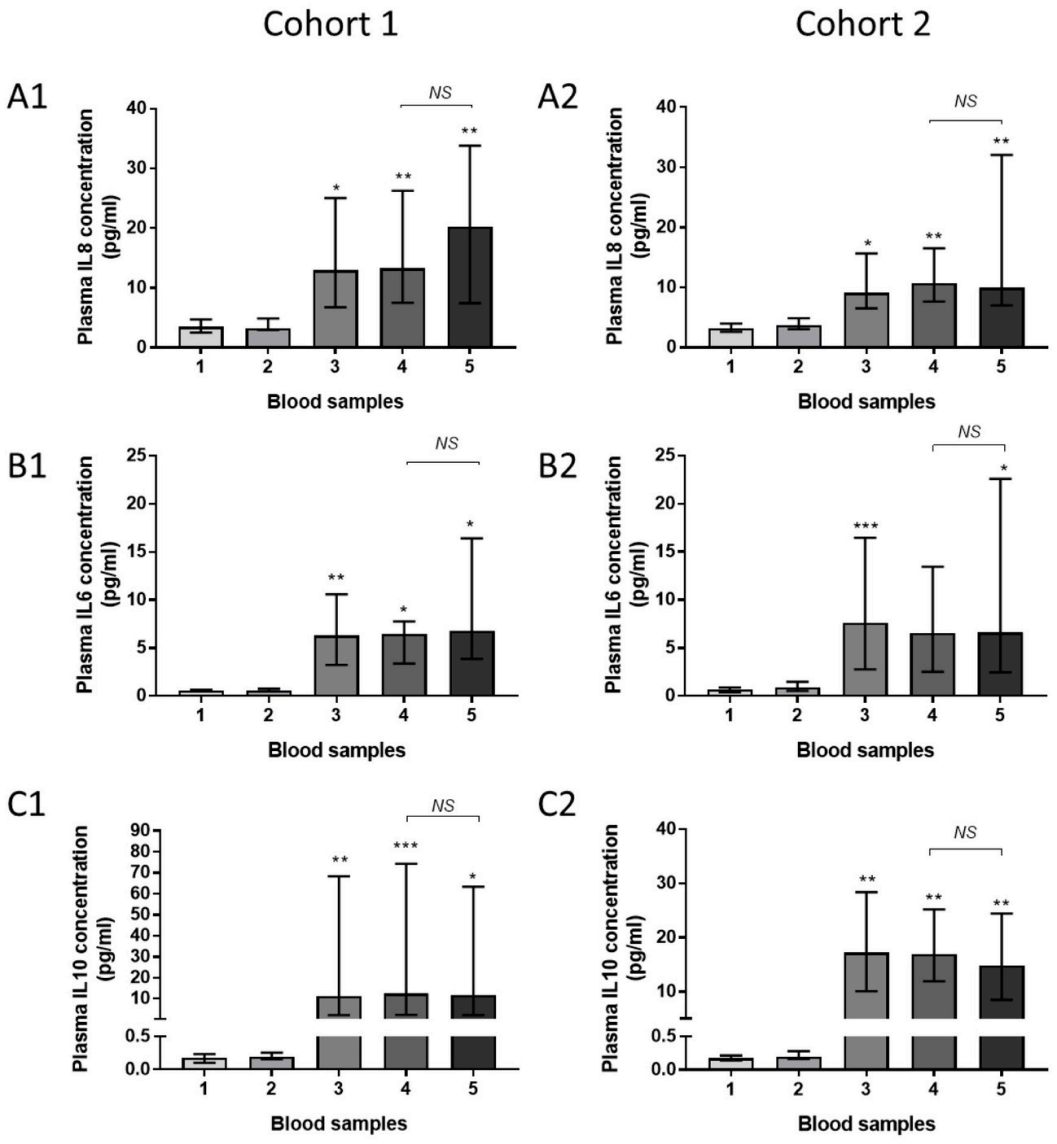
Plasma cytokines. Data are presented as median (columns) and interquartile range (bars). Left-sided graphs represent cohort 1, right-sided cohort 2. [A1,2] IL-8, [B1, 2] IL-6 and [C1,2] IL-10. The Friedman test with Dunn’s post-hoc multiple comparisons compared values between the sample 1 and samples 2–5. Wilcoxon’s matched pairs signed rank test compared samples 4 and 5. *p<0.05, **p<0.005, ***p<0.001. Abbreviations NS: non-significant (Cohort 1: n = 8 Cohort 2: n = 6).

Significantly higher plasma concentrations of IL-8, IL-6 and IL-10 were observed in post-CPB samples compared to pre-CPB samples in both cohorts (Fig 4). No significant differences were observed in pulmonary vein plasma cytokine concentrations between the right (ventilated) and left (deflated) lung following CPB. Concentrations of TNF-α, IL-1β, IL-12p70, IL-2, IL-4, IL-13 and interferon gamma remained similar across blood samples (supporting information section S3 and S4 Figs).

### Clinical outcomes

**Fig 5:**
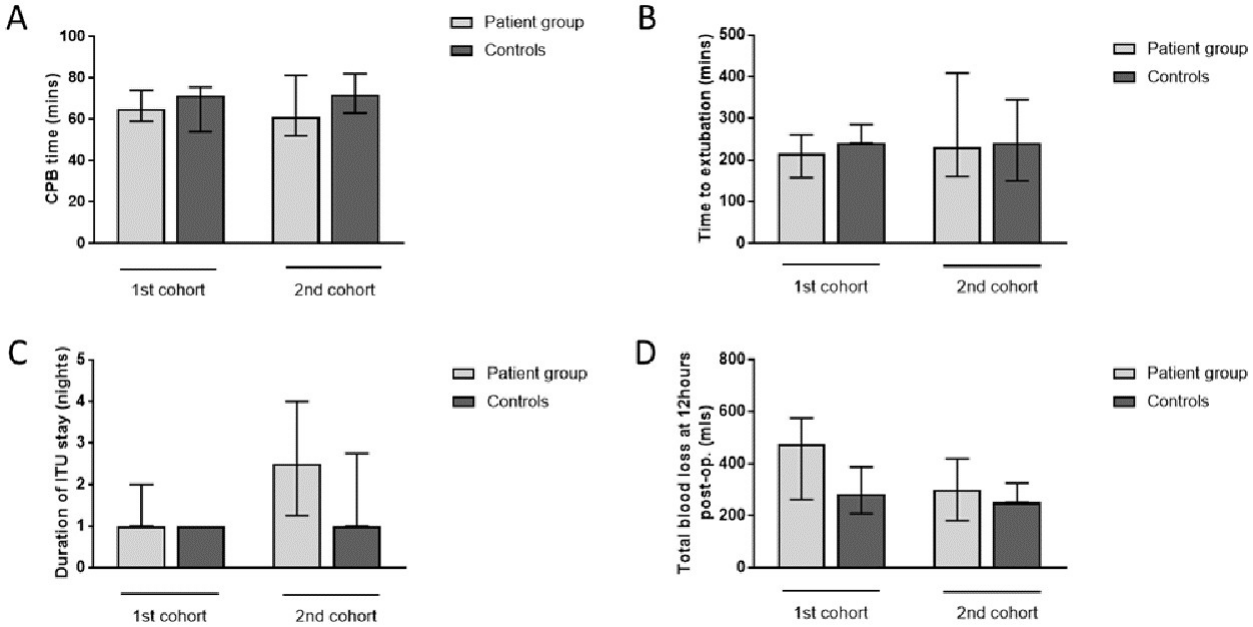
Clinical outcome measures. [A] Total CPB time. [B] Time to extubation. [C] Duration of ICU stay. [D] Total blood loss (total volume in mediastinal and pleural drains at 12 hours post-operatively). Data are presented as median (columns) and interquartile range (bars). The control groups consisted of age- and sex-matched patients who underwent first time CABG procedures in the same period as patients recruited to the respective study cohorts. Mann-Whitney U test was used to compare the patient and control groups. Cohort 1: patient group n = 9, controls n = 8, Cohort 2: patient group n = 8, controls n = 7 [A] and n = 8 [B, C, D].

Lung ventilation during CPB had no significant impact on pulmonary vein blood gas measurements (supporting information section, S2 and S3 Tables). OLV during CPB had no clear impact on measures of total CPB time, time to extubation, duration of ICU stay, or total blood loss from mediastinal and pleural drains relative to age- and sex-matched controls (Fig 5 and supporting information section S4 Table). Chest radiograph reports revealed no obvious differences between the groups (supporting information section S5 Table).

## Discussion

Summers and colleagues elegantly demonstrated that the healthy human lung can retain primed neutrophils, facilitate their de-priming and subsequently release them into the systemic circulation [3, 4]. Our preliminary data suggest deflation of the lung during CPB was not associated with alterations in the important neutrophil functions of phagocytosis or priming. Altering the degree of lung inflation in the two cohorts did not alter phagocytosis or priming status.

Our data also highlight the potential for intra-operative right and left pulmonary vein sampling to assess how altered lung properties (in this case comparing a deflated and inflated lung) may influence blood returning to the left atrium. The model could be readily adjusted to other interventions—for example inhaled medication could be delivered to one lung, with both lungs inflated to the same degree, and lung protective effects assessed. Lung-protective inhaled therapy has been described in rodents [17]. Bronchoalveolar lavage of each lung could readily be incorporated into the model, extending its usefulness.

Our cohorts were too small to draw meaningful conclusions relating to clinical outcomes or safety of the model. However, no clear adverse safety signals were attributable to the model in the small numbers studied.

### Comparison with other studies

To our knowledge no other studies have specifically performed bilateral pulmonary vein sampling to assess human neutrophil function, and so comparisons relate mostly to samples 1, 2 and 3 in our cohorts.

Little is known about the impact of CABG with CPB on neutrophil phagocytic function, despite the key role of neutrophil phagocytosis in preventing and clearing bacterial infection. Previous studies reported varying results, some demonstrating preserved phagocytic capacity throughout surgery with CPB [18–20], others reported transient deficiency [21, 22].

Neutrophil priming has been implicated in post-CPB lung injury based on the concept that primed neutrophils pool in the pulmonary vasculature [23–25]. Previous studies have identified post-CPB neutrophil priming through an up-regulation of CD11b on circulating neutrophils [23, 26–28], and/or a down-regulation of CD62L [27, 29]. In contrast, our results are in keeping with studies showing no alteration in cell surface adhesion makers following CPB [30, 31].

The previously described increase in plasma concentrations of the pro-inflammatory cytokines IL-8 and IL-6 and the anti-inflammatory cytokine IL-10 was observed in all post-operative blood samples, as expected.

### Methodological considerations and limitations

A key limitation of the study is the small sample size. The study was designed to describe a model of intra-operative, bilateral pulmonary vein sampling as well as the effects of lung deflation/inflation on neutrophil functions, and the cohorts were sufficient to achieve these objectives. However, the cohorts were too small to reach firm conclusions on other outcomes. In particular, no inferences can be drawn on the effect of OLV on important clinical outcomes in this study. In this regard our secondary objective was solely to exclude any obvious adverse safety signals in using the model.

Secondly, while we could model lung deflation versus inflation, and while we could control tidal volume in the two cohorts, it is important to recognize that several other ventilatory parameters can influence pulmonary and systemic inflammation during CPB, including respiratory rate, PEEP, plateau pressure and peak pressure. These parameters were not assessed here, but the model is amenable to their incorporation in future studies. Furthermore, we used a different FiO_2_ in each cohort.

A technical limitation was that, under ideal conditions, the post-CPB central and pulmonary venous samples would be taken immediately upon simultaneous restoration of bilateral mechanical ventilation and full circulation, after discontinuation of CPB. This is difficult to achieve due to practical considerations such as the initiation of bilateral lung ventilation prior to weaning the patient fully off CPB. We believe sampling times (supporting information section, S1 Table) were close to the shortest practically achievable. However, we cannot exclude the possibility that a beneficial effect of lung ventilation on neutrophil function was lost in the few minutes before sampling.

Another technical limitation relates to whether OLV provided sufficient lung expansion to achieve putative benefits in neutrophil function. We chose two levels of ventilation to determine whether there may be a dose-response in neutrophil function, but cannot exclude the possibility that an optimal level of lung expansion was missed. Moreover, we did not collect data on intraoperative transfusion of blood or blood products and there remains the theoretical possibility that this may have influenced the results obtained between the pre and post bypass blood samples. In addition, while we used the same model of CPB circuit, pump and oxygenator for all study patients to ensure methodological consistency, the use of different models in other institutions may affect the generalizability of our results.

From the perspective of neutrophil function, we cannot exclude potential sampling bias, as it is theoretically possible that primed or dysfunctional neutrophils were retained in the pulmonary microvasculature, and only “healthy” neutrophils released into the pulmonary veins. Addressing this potential limitation would have required more invasive investigations such as intra-operative radionuclide studies or lung biopsy, which we did not consider justifiable.

A further limitation relates to the delay in processing pre-CPB samples, given that all 5 samples were kept in a cool box and processed simultaneously to ensure consistent processing of samples. However, data from our own and other laboratories suggest that the only assay to be potentially influenced by this variation is the apoptosis assay. Finally, we did not cover all neutrophil functions, and did not study other immune cells.

### Conclusion

In conclusion, an OLV model with direct intra-operative, bilateral pulmonary vein sampling creates opportunities to assess interventions applied to one lung in a controlled manner. In our hands, the model suggested key neutrophil functions are similar after passage through a deflated or inflated lung.

## Supporting information

S1 Protocol Ventilation and blood sampling protocols for cohort 1 and cohort 2. (DOCX)

S1 Fig Consort diagram for cohort 1. †Reasons for exclusion: surgeon not participating in study (n = 29), operation on afternoon list (n = 10), immunosuppressant drugs/immunosuppressed (n = 4), listed too late for consent (n = 2), possible simultaneous valve replacement (n = 1). ‡ Reasons eligible patients not recruited: operation cancelled/postponed after written consent (n = 4), operation cancelled/postponed before written consent (n = 4), scheduled at same time as another potential study patient (n = 4), clinical commitments of research fellow (n = 3), declined (n = 1).
(TIF)

S2 Fig Consort diagram demonstrating patient screening and recruitment to cohort 2. †Reasons for exclusion: surgeon not participating in study (n = 42), operation on afternoon list (n = 11), listed too late for consent (n = 5). ‡Reasons eligible patients not recruited: operation cancelled/postponed before written consent (n = 5), scheduled at same time as another study patient (n = 2), clinical commitments of research fellow (n = 8).
(TIF)

S3 Fig Plasma concentrations of TNFα [A], IL-1β [B], IL-12p70 [C], IL-2 [D], IL-4 [E], IL-13 [F] and IFNγ [G] across all five blood samples in cohort 1. Data are presented as median (columns) and interquartile range (bars). Blood samples 1–5 are as described in the Materials and Methods section. The Friedman test with Dunn’s post-hoc multiple comparisons was used for comparing values between the baseline blood sample (sample 1) and samples 2–5. The Wilcoxon matched pairs signed rank test was used to evaluate differences between the right, ventilated lung (sample 4) and the left, deflated lung (sample 5). *p<0.05. Abbreviations NS: non-significant. N = 8 for TNFα and IFNγ, n = 7 for IL-1β, IL-12p70 and IL-2 and n = 6 for IL-4 and IL-13.
(TIFF)

S4 Fig Plasma concentrations of TNFα [A], IL-1β [B], IL-12p70 [C], IL-2 [D], IL-13 [E] and IFNγ [F] across all five blood samples in cohort 2. (Data for IL-4 not shown due to multiple undetectable levels). Data are presented as median (columns) and interquartile range (bars). Blood samples 1–5 are as described in the Materials and Methods section. The Friedman test with Dunn’s post-hoc multiple comparisons was used for comparing values between the baseline blood sample (sample 1) and samples 2–5. The Wilcoxon matched pairs signed rank test was used to evaluate differences between the right, ventilated lung (sample 4) and the left, deflated lung (sample 5). Abbreviations NS: non-significant. N = 6 for TNFα, IL-1β, IL-12p70, IL-2 and IFNγ and n = 4 for IL-13.
(TIFF)

S1 Table Operative data for the first and second cohorts. Data are presented as median (interquartile range), n = 9 first cohort, n = 8 second cohort. † Post-CPB blood samples refer to samples 3, 4 and 5 which were all obtained following cessation of cardiopulmonary bypass.
(TIFF)

S2 Table Pulmonary vein blood gas parameters between the left (deflated lung) and right (ventilated lung) following resumption of pulmonary blood flow after CPB in cohort 1. Data are presented as median (interquartile range). Statistical analysis was by the Wilcoxon matched-pairs signed rank test. N = 8. A statistically significant increase in PCO_2_ was observed in blood from the right pulmonary vein compared to the left pulmonary vein however PCO_2_ values for both pulmonary veins remained within normal limits.
(TIFF)

S3 Table Pulmonary vein blood gas parameters between the left (deflated lung) and right (ventilated lung) following resumption of pulmonary blood flow after CPB in cohort 2. Data are presented as median (interquartile range). Statistical analysis was by the Wilcoxon matched -pairs signed rank test. N = 5.
(TIFF)

S4 Table Comparison of the baseline clinical characteristics for cohort 1 and 2 patients and their respective age and sex-matched control groups. Data are presented as median (interquartile range). Statistical analysis was by the Mann-Whitney U-test. *n = 7 (no available height value for 1 patient).
(TIFF)

S5 Table Classification of the post-operative mobile chest radiograph report for cohort 1 and 2 patients and their respective age and sex-matched control groups. (TIFF)
