# Supplementary material for: A human model of bilateral pulmonary vein sampling to assess the effects of one-lung ventilation on neutrophil function
Source: PLoS One. 2022 Jul 26;17(7):e0271958. doi: 10.1371/journal.pone.0271958 (PMC9321419; doi:10.1371/journal.pone.0271958)
Supplement: S1 Protocol — (DOCX) [file pone.0271958.s001.docx]

## **S1 Protocol: Ventilation and blood sampling protocols for cohort 1 and cohort 2**

### Cohort 1:

1. Patient anaesthetized, intubated and bilateral ventilation commenced with standardized settings as follows:
2. Volume-controlled ventilation
3. FiO_2_ to maintain SaO_2_ between 96-99%
4. Tidal volume: 6-8 mL kg^-1^ of ideal body weight
5. Airway pressure: <25 cmH_2_O
6. PEEP: 5-8 cmH_2_O
7. 10-12 ventilations per minute.
8. Central venous line placed in internal jugular vein. Blood sample 1 obtained (25mls of venous blood from the central line) by the anesthetist. The first 3mls of blood was discarded to account for residual flush in the line and this was the case for all subsequent central vein samples.
9. Bronchus-blocking balloon placed in the left main bronchus following intubation and the position of the balloon confirmed by fiber-optic bronchoscopy by the anesthetist. However, the balloon was not inflated at this point. Bilateral ventilation continued as per the standardized ventilation settings at this point.
10. Surgery commenced.
11. Just before intravenous heparin given, blood sample 2 obtained (25mls of venous blood from central line) by the anesthetist.
12. The left pleural cavity was confirmed as opened by the surgeon (a small incision was made in the left pleura by the surgeon if not opened when accessing the left internal mammary artery: this was to achieve maximal collapse of the left lung when bilateral ventilation was stopped).
13. Patient placed on bypass and at the time when ventilation is normally discontinued:
14. Both lungs deflated
15. Surgeon confirms when left lung is fully deflated
16. Left bronchus-blocking balloon inflated
17. Ventilation to the right lung commenced with standardized settings as follows:
18. Volume-controlled ventilation
19. FiO_2_: 0.21
20. Tidal volume: 2 mL kg^-1^ of ideal body weight
21. 7 ventilations per minute
22. PEEP: 5cmH_2_O.
23. CABG procedure commenced on CPB.
24. CABG completed.
25. Both lungs re-ventilated as per the following standardized settings after deflation of the left bronchus-blocking balloon:
26. Volume-controlled ventilation
27. FiO_2_ at discretion of anesthetist to maintain SaO2 96-99%
28. Tidal volume: 6-8 mL kg^-1^ of ideal body weight
29. Airway pressure: <25cmH_2_O
30. PEEP: 5-8 cmH_2_O
31. 10-12 ventilations per minute.
32. Patient weaned off bypass (approximately 5 minutes after bilateral ventilation re-commenced).
33. Intravenous protamine given after patient weaned off bypass.
34. Two minutes after protamine given, 3 blood samples taken before chest closure as follows:
35. 25 mL blood sample (sample 3) from central line taken by the anesthetist
36. 25 mL blood sample (sample 4) from a right pulmonary vein taken by the surgeon with 21-gauge needle and 30 mL syringe
37. 25 mL blood sample (sample 5) from a left pulmonary vein taken by the surgeon with 21-gauge needle and 30 mL syringe.

NB: The surgeon was advised on whether to sample the left or right pulmonary vein first: alternate sides for each study participant.

1. Normal chest closure
2. At the end of the procedure all labelled samples (1-5) were immediately transported to the laboratory for further analysis.

## Cohort 2:

For cohort 2, the ventilation protocol differed from cohort 1 in two principal ways. Firstly, for one-lung ventilation during CPB, the FiO_2_ administered to the right lung was increased to 0.5 (compared to 0.21 in cohort 1). Secondly, for one-lung ventilation during CPB, the tidal volume ventilation to the right lung was increased to 4 mL kg^-1^ ideal body weight (compared to 2 mL kg^-1^ ideal body weight in cohort 1). All other aspects of the ventilation and blood sampling protocol remained the same as for cohort 1.

## 
