# Supplementary figures and images for: A human model of bilateral pulmonary vein sampling to assess the effects of one-lung ventilation on neutrophil function
Source: PLoS One. 2022 Jul 26;17(7):e0271958. doi: 10.1371/journal.pone.0271958 (PMC9321419; doi:10.1371/journal.pone.0271958)

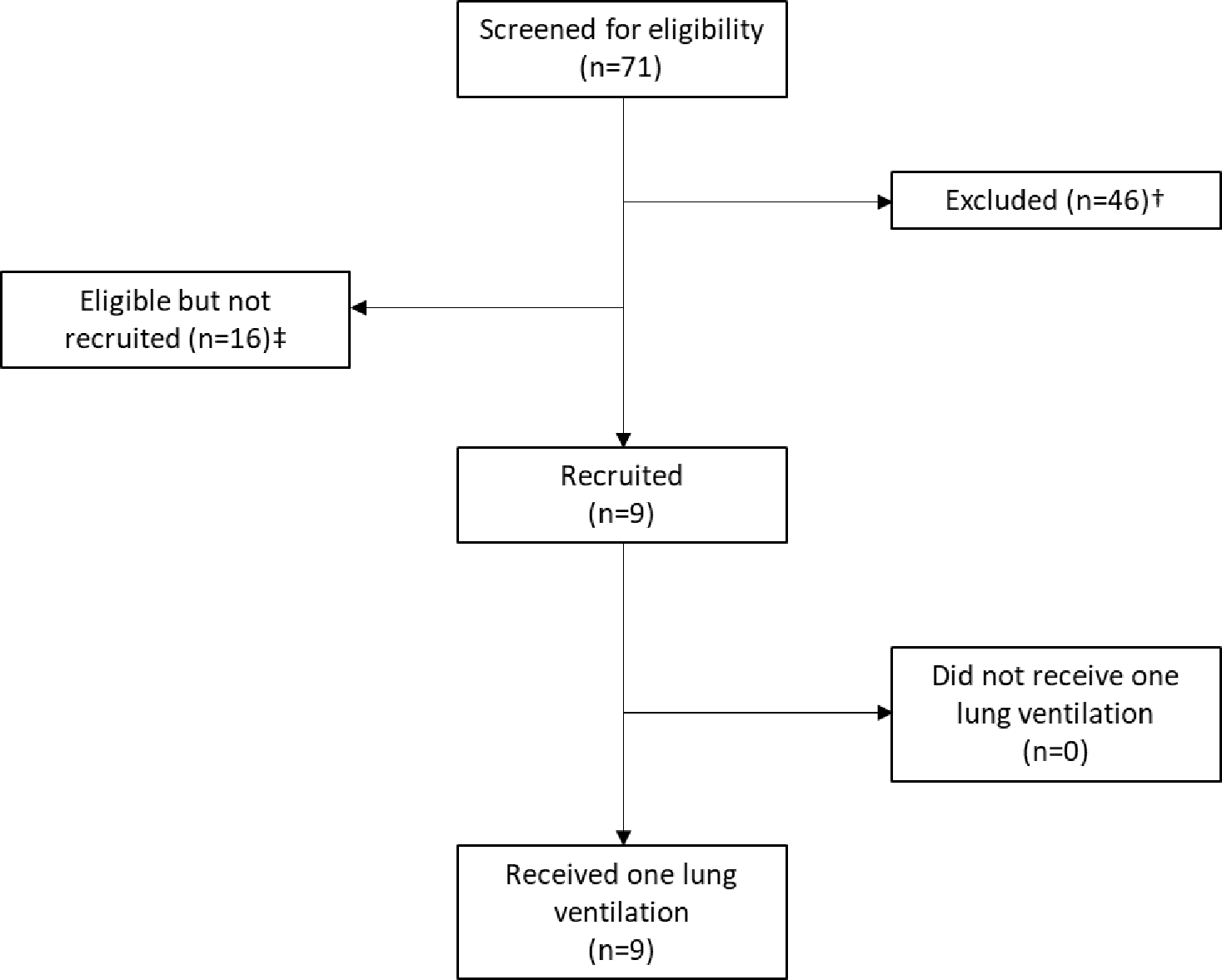

Supplement: S1 Fig — †Reasons for exclusion: surgeon not participating in study (n = 29), operation on afternoon list (n = 10), immunosuppressant drugs/immunosuppressed (n = 4), listed too late for consent (n = 2), possible simultaneous valve replacement (n = 1). ‡ Reasons eligible patients not recruited: operation cancelled/postponed after written consent (n = 4), operation cancelled/postponed before written consent (n = 4), scheduled at same time as another potential study patient (n = 4), clinical commitments of research fellow (n = 3), declined (n = 1). (TIF) [file pone.0271958.s002.tif]

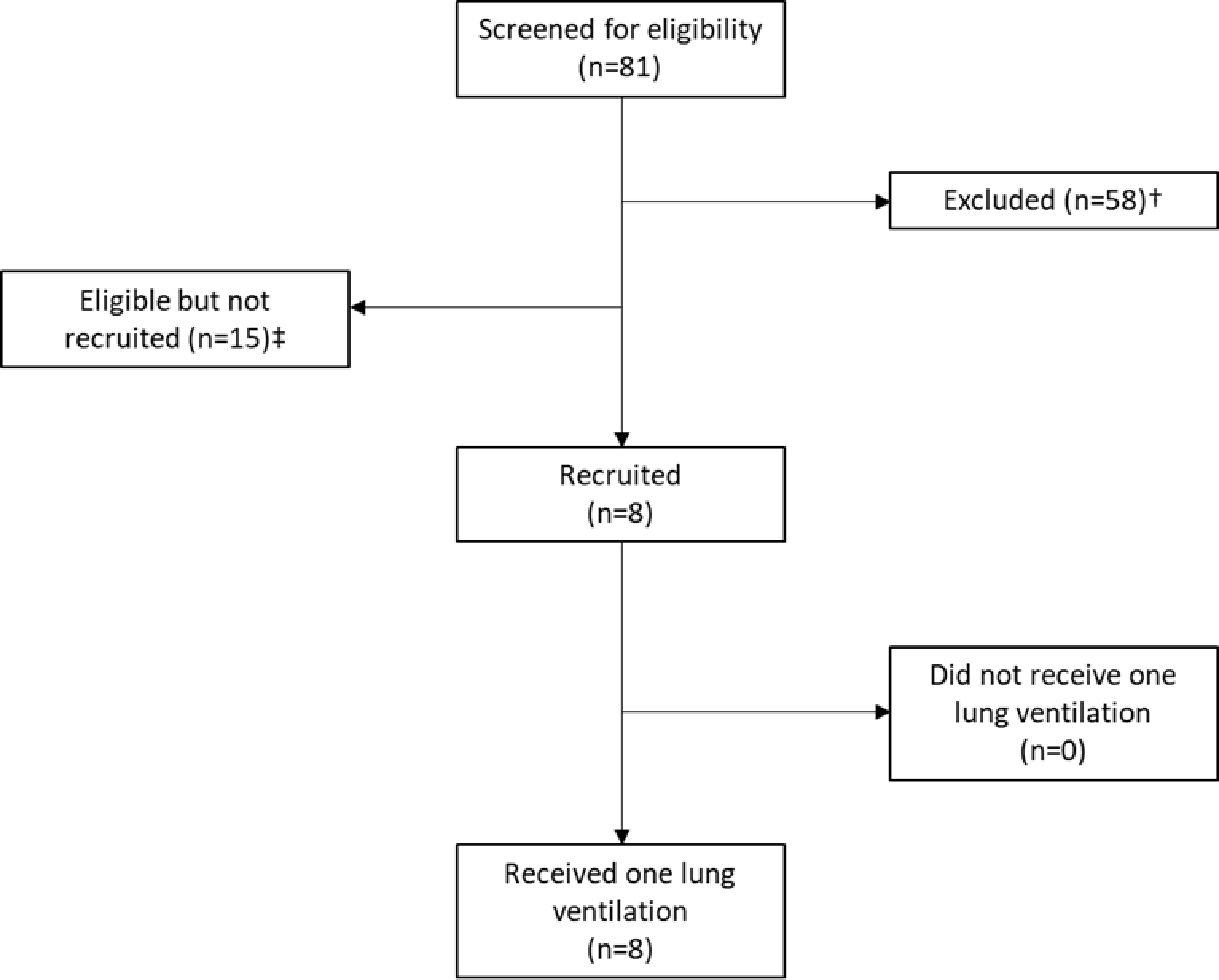

Supplement: S2 Fig — †Reasons for exclusion: surgeon not participating in study (n = 42), operation on afternoon list (n = 11), listed too late for consent (n = 5). ‡Reasons eligible patients not recruited: operation cancelled/postponed before written consent (n = 5), scheduled at same time as another study patient (n = 2), clinical commitments of research fellow (n = 8). (TIF) [file pone.0271958.s003.tif]

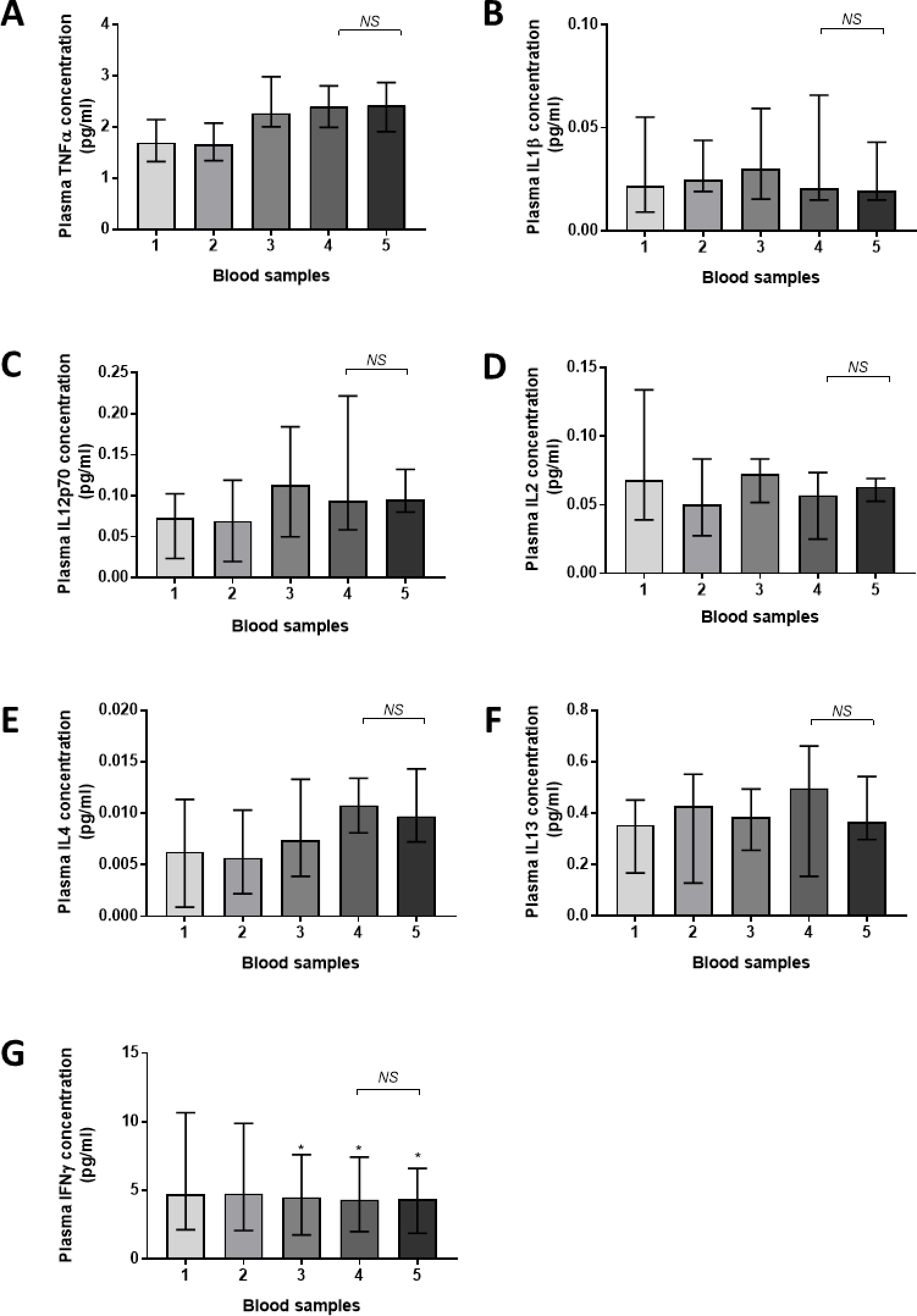

Supplement: S3 Fig — Data are presented as median (columns) and interquartile range (bars). Blood samples 1–5 are as described in the Materials and Methods section. The Friedman test with Dunn’s post-hoc multiple comparisons was used for comparing values between the baseline blood sample (sample 1) and samples 2–5. The Wilcoxon matched pairs signed rank test was used to evaluate differences between the right, ventilated lung (sample 4) and the left, deflated lung (sample 5). *p<0.05. Abbreviations NS: non-significant. N = 8 for TNFα and IFNγ, n = 7 for IL-1β, IL-12p70 and IL-2 and n = 6 for IL-4 and IL-13. (TIFF) [file pone.0271958.s004.tiff]

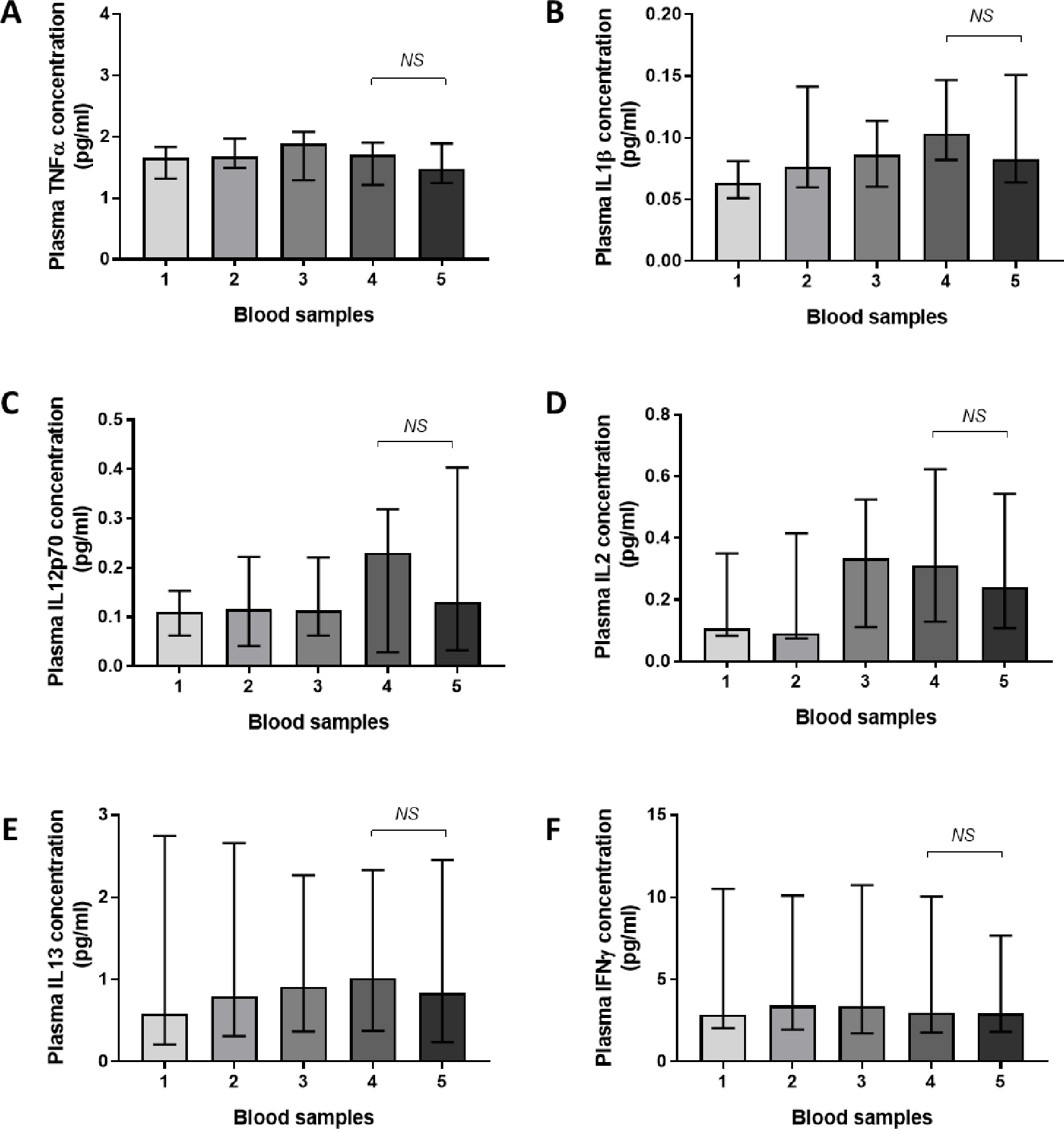

Supplement: S4 Fig — (Data for IL-4 not shown due to multiple undetectable levels). Data are presented as median (columns) and interquartile range (bars). Blood samples 1–5 are as described in the Materials and Methods section. The Friedman test with Dunn’s post-hoc multiple comparisons was used for comparing values between the baseline blood sample (sample 1) and samples 2–5. The Wilcoxon matched pairs signed rank test was used to evaluate differences between the right, ventilated lung (sample 4) and the left, deflated lung (sample 5). Abbreviations NS: non-significant. N = 6 for TNFα, IL-1β, IL-12p70, IL-2 and IFNγ and n = 4 for IL-13. (TIFF) [file pone.0271958.s005.tiff]

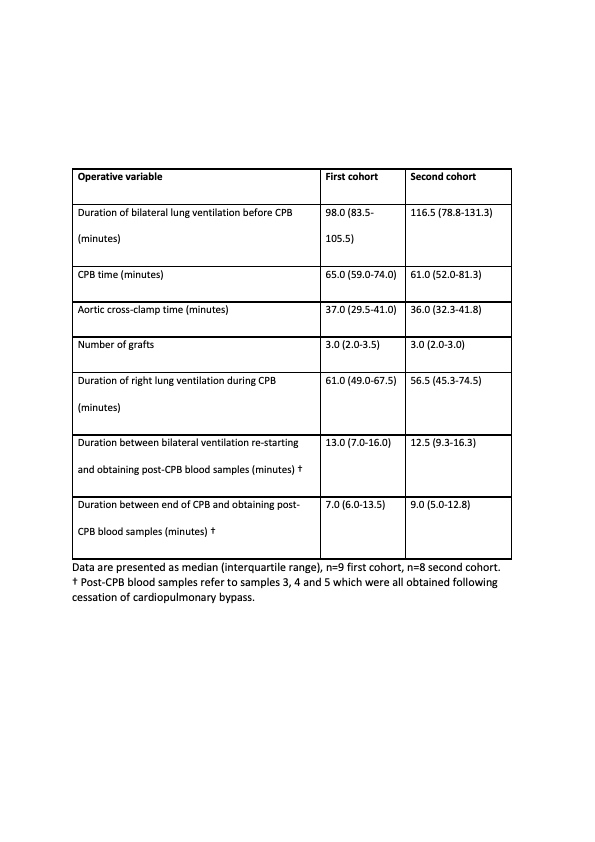

Supplement: S1 Table — Data are presented as median (interquartile range), n = 9 first cohort, n = 8 second cohort. † Post-CPB blood samples refer to samples 3, 4 and 5 which were all obtained following cessation of cardiopulmonary bypass. (TIFF) [file pone.0271958.s006.tiff]

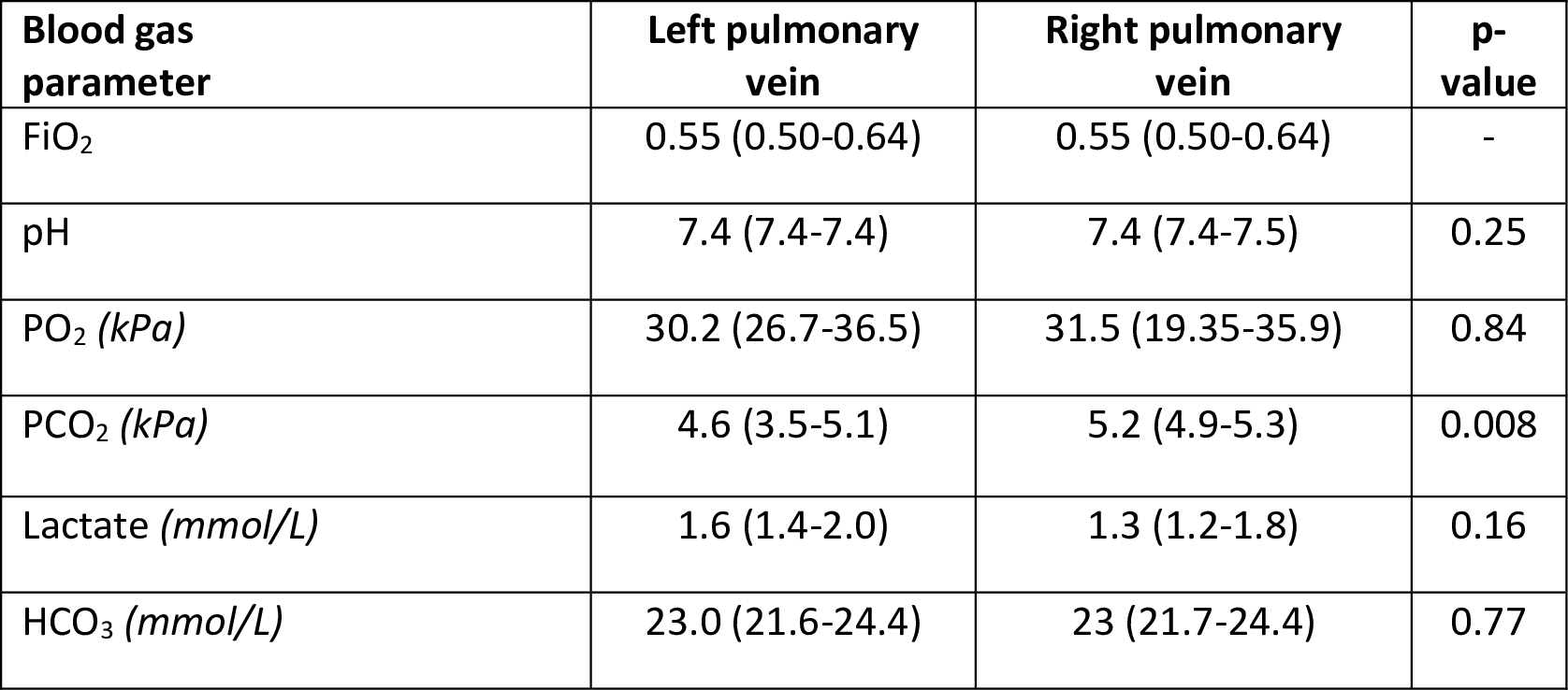

Supplement: S2 Table — Data are presented as median (interquartile range). Statistical analysis was by the Wilcoxon matched-pairs signed rank test. N = 8. A statistically significant increase in PCO2 was observed in blood from the right pulmonary vein compared to the left pulmonary vein however PCO2 values for both pulmonary veins remained within normal limits. (TIFF) [file pone.0271958.s007.tiff]

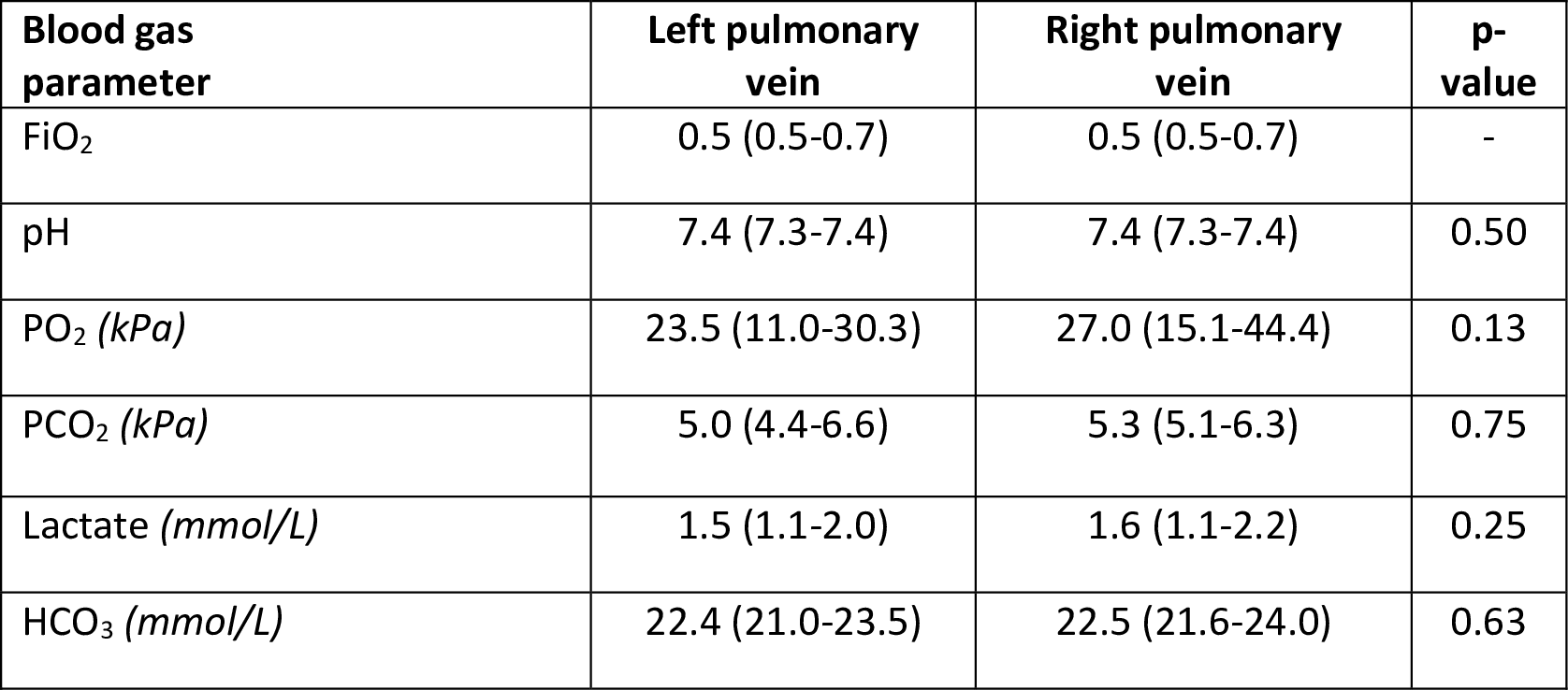

Supplement: S3 Table — Data are presented as median (interquartile range). Statistical analysis was by the Wilcoxon matched -pairs signed rank test. N = 5. (TIFF) [file pone.0271958.s008.tiff]

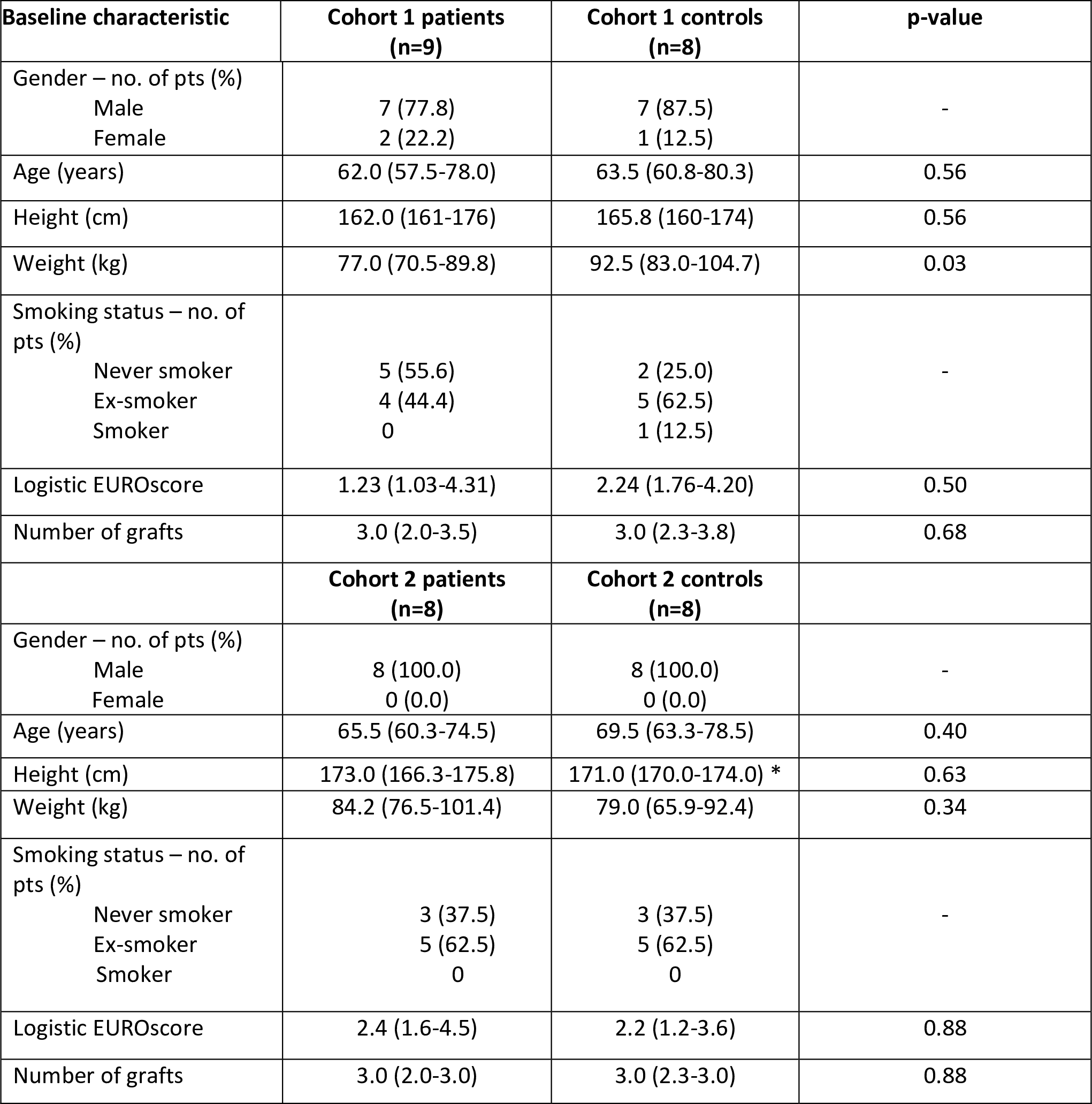

Supplement: S4 Table — Data are presented as median (interquartile range). Statistical analysis was by the Mann-Whitney U-test. *n = 7 (no available height value for 1 patient). (TIFF) [file pone.0271958.s009.tiff]

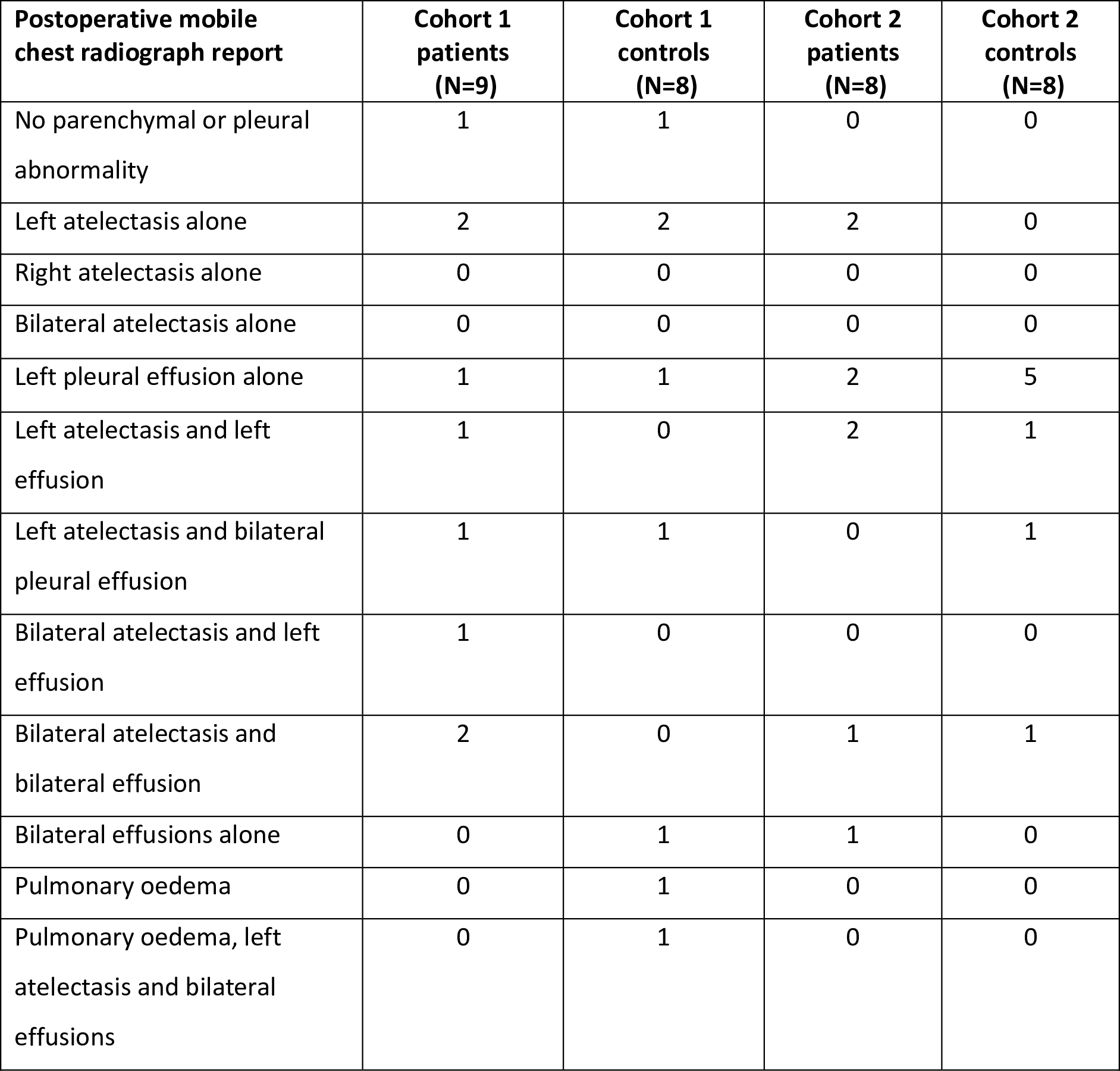

Supplement: S5 Table — (TIFF) [file pone.0271958.s010.tiff]
